# Towards resolution of the intron retention paradox in breast cancer

**DOI:** 10.1186/s13058-022-01593-1

**Published:** 2022-12-29

**Authors:** Jaynish S. Shah, Michael J. G. Milevskiy, Veronika Petrova, Amy Y. M. Au, Justin J. L. Wong, Jane E. Visvader, Ulf Schmitz, John E. J. Rasko

**Affiliations:** 1 Computational BioMedicine Laboratory Centenary Institute, The University of Sydney, Camperdown, Australia; 2 Gene and Stem Cell Therapy Program Centenary Institute, The University of Sydney, Locked Bag No. 6, Newtown, NSW 2042 Australia; 3 Australian Centre for Blood Diseases, Central Clinical School, Monash University and Alfred Health, Melbourne, VIC Australia; 4 ACRF Cancer Biology and Stem Cells Division, The Walter and Eliza Hall Institute of Medical Research, Parkville, VIC 3052 Australia; 5 Epigenetics and RNA Biology Program Centenary Institute, The University of Sydney, Camperdown, 2050 Australia; 6 Faculty of Medicine and Health, The University of Sydney, Camperdown, Australia; 7 Department of Medical Biology, The University of Melbourne, Parkville, VIC 3010 Australia; 8 Department of Molecular and Cell Biology, College of Public Health, Medical and Veterinary Sciences, James Cook University, 1 James Cook Drive, Townsville, QLD 4811 Australia; 9 Centre for Tropical Bioinformatics and Molecular Biology, Australian Institute of Tropical Health and Medicine, James Cook University, Cairns, 4878 Australia; 10 Cell and Molecular Therapies, Royal Prince Alfred Hospital, Camperdown, Australia

**Keywords:** Alternative splicing, Patient stratification, Luminal B breast cancer, Adipocytes, Cancer transcriptomics

## Abstract

**Background:**

After many years of neglect in the field of alternative splicing, the importance of intron retention (IR) in cancer has come into focus following landmark discoveries of aberrant IR patterns in cancer. Many solid and liquid tumours are associated with drastic increases in IR, and such patterns have been pursued as both biomarkers and therapeutic targets. Paradoxically, breast cancer (BrCa) is the only tumour type in which IR is reduced compared to adjacent normal breast tissue.

**Methods:**

In this study, we have conducted a pan-cancer analysis of IR with emphasis on BrCa and its subtypes. We explored mechanisms that could cause aberrant and pathological IR and clarified why normal breast tissue has unusually high IR.

**Results:**

Strikingly, we found that aberrantly decreasing IR in BrCa can be largely attributed to normal breast tissue having the highest occurrence of IR events compared to other healthy tissues. Our analyses suggest that low numbers of IR events in breast tumours are associated with poor prognosis, particularly in the luminal B subtype. Interestingly, we found that IR frequencies negatively correlate with cell proliferation in BrCa cells, i.e. rapidly dividing tumour cells have the lowest number of IR events. Aberrant RNA-binding protein expression and changes in tissue composition are among the causes of aberrantly decreasing IR in BrCa.

**Conclusions:**

Our results suggest that IR should be considered for therapeutic manipulation in BrCa patients with aberrantly low IR levels and that further work is needed to understand the cause and impact of high IR in other tumour types.

**Supplementary Information:**

The online version contains supplementary material available at 10.1186/s13058-022-01593-1.

## Background

Pre-mRNA splicing is a ubiquitous process that is crucial for the maintenance of transcriptomic complexity and gene expression regulation in eukaryotic cells [1, 2]. Perturbations to this highly calibrated system can have severe consequences and lead to diseases including cancer [3–6]. In this context, numerous studies describing intron retention (IR) in disease have shed light on the mechanisms leading to aberrant and pathological IR [7–9].

The importance of IR in cancer has been emphasized following landmark discoveries about (i) aberrant IR patterns in leukaemia [10, 11], (ii) IR as a source of neoepitopes [12], (iii) tumour suppressor gene inactivation by intronic polyadenylation [13], (iv) IR-based biomarkers [14, 15], and (v) IR as a therapeutic target [16].

IR is regulated by *cis*- and *trans*-acting modulators [2, 17, 18] facilitating cellular responses to a range of environmental stimuli [19]. Intron-retaining mRNA transcripts are often degraded via nonsense-mediated decay (NMD), thereby causing downregulation of the host gene. The burden of IR in disease is governed by perturbations to mechanisms known to regulate this form of alternative splicing, including mutations, splicing factor dysregulation, and epigenetic variations.

However, despite the cumulative evidence for the importance of IR in cancer, a systematic analysis of IR regulation in breast cancer (BrCa) and the role of aberrant IR in BrCa biology has not been conducted to date. In this study, we sought to resolve the paradox wherein breast cancer exhibits reduced IR, which is an important consequence of alternative splicing.

We analysed 615 BrCa patient transcriptomes which included four major molecular subtypes (Luminal A, Luminal B, Basal, and Her2 positive). We confirmed a consistent downregulation of IR in BrCa. However, we also observed that normal breast tissue has a significantly higher IR event frequency compared to other healthy tissues. The number of IR events correlated with survival in the luminal B BrCa subtype. Differences in IR frequencies are largely influenced by the tissue’s cellular composition as well as specific dysregulated RNA-binding proteins (RBPs).

## Methods

### RNA-sequencing data/patient samples

We retrieved data from nine tumour types and healthy adjacent tissue, including 615 BrCa patient samples generated by the TCGA. We used TCGA metadata to assign the samples to molecular subtypes (i.e. Luminal A, Luminal B, human epidermal growth factor receptor 2 (HER2)-enriched, and Basal-like) based on the PAM50 classification system. On one occasion (Additional file 1: Fig. S2C), we grouped samples based on the immunohistochemical (IHC) score for HER2.

Only samples for which sequencing had been performed at > 40 M read depth were selected for analysis. Moreover, only tumour types with at least 20 matched tumour/normal tissue samples were considered. RNA-seq data were downloaded as BAM files using the R/Bioconductor package TCGAbiolinks [20] and the command-line tool gdc-client v1.4.0 (github.com/NCI-GDC/gdc-client) under an approved data access application. All files were checked for integrity. Harmonized gene expression data in the form of HTseq counts (RRID:SCR_005514) [21] were downloaded using TCGAbiolinks (RRID:SCR_017683).

### mRNA-sequencing and data analysis—MCF7 and MCF10A cells

Total RNA was isolated from MCF7 and MCF10A cells using TRIzol (Invitrogen). The RNA quality was assessed using RNA 6000 Nano Chips on an Agilent Bioanalyzer (Agilent Technologies) to confirm an RNA integrity score of > 7.0. mRNA-seq was performed by Macrogen (Korea; RRID:SCR_014454) using the Illumina Hi-Seq 2000 platform. RNA-seq libraries were prepared from > 1 μg of total RNA using TruSeq RNA sample prep kit (Illumina) according to the manufacturers’ instructions.

### Differential IR and gene expression analyses

IR was quantified using IRFinder v1.2.0 [17], using the Ensembl human genome (hg38, release 86; RRID:SCR_002344) as reference. The IRFinder algorithm measures 20 parameters for IR detection in each sample, including the median number of reads mapping to each nucleotide across the intron length (intron depth, ID), the ratio of nucleotides within an intron with mapped reads (coverage), the number of reads that map to the 5′ flanking exon and to another exon within the same gene (splice left, SL), the number of reads that map to the 3′ flanking exon and to another exon within the same gene (splice right, SR), the number of reads spanning the exon-exon junction (splice exact, SE) as well as the IR ratio:

$$\frac{\mathrm{ID}}{\mathrm{ID}+\mathrm{ max}(\mathrm{SL},\mathrm{ SR})}$$

Some selection criteria for IR events were chosen to minimize the chance of false positive IR calling while at the same time maintaining sufficient sensitivity to avoid too many false negative events. The following criteria were used for quantifying the number of IR events in a sample:

1. $$0.7\le \frac{\mathrm{SL}}{SR}\le 1.3;$$
  This filter ensures that introns are flanked by constitutive exons.
2. $(\mathrm{SL}+\mathrm{SR})>10$ in ≥ 50% of samples;
  Flanking exons need to be well expressed in most samples to avoid false positive IR events.
3. $\mathrm{coverage}>0.5$ in ≥ 50% of samples;
  Only introns with extensive coverage in the majority of samples are considered to prevent confounding factors that could lead to false IR calling.
4. $\mathrm{IR}>0.05$ in ≥ 50% normal or cancer samples.
  We included only samples with at least 40M reads to facilitate accurate IR quantification. We considered a 5% inclusion rate for IR to be of biological relevance.

The number of IR events in a sample was determined based on introns with an IR ratio > 0.1 and meeting the filtering criteria described above. Introns not meeting these criteria were not considered as being retained. Beta regression was used to identify differentially retained introns (dIR) between cancer and adjacent normal tissues using the betareg R package [22]. Since IR ratios are proportional data with values between 0 and 1, we reasoned that beta regression was best suited to model IR and identify dIRs between normal and cancer tissues. An absolute difference in the IR ratio (ΔIR = IR_Cancer_ − IR_Normal_) of more than 0.1 with FDR-adjusted *p* < 0.05 was considered significant.

Dimensionality reduction, i.e. principal component analysis (PCA), of IR profiles was performed using the package factoextra (github.com/kassambara/factoextra).

Differential gene expression between normal breast tissue and BrCa was performed using the DESeq2 package (RRID:SCR_000154) [23]. Genes with an average read count > 10 in all samples were selected for differential gene expression analysis (*n* = 23,072). Genes with an absolute log2 fold change > 1 and FDR-adjusted *p* < 0.05 were considered significant. To identify genes that were specifically differentially expressed in BrCa, we removed genes that were differentially expressed in any of the other 8 cancers and determined specificity by computing the z-score on log fold change using the log fold change observed in BrCa as reference.

### Gene Ontology and RBP analyses

Gene Ontology analysis was performed using the clusterProfiler package (RRID:SCR_016884) [24]. The false discovery rate (FDR) approach was used for multiple testing correction. The list of 1542 RBPs was taken from Gerstberger et al*.* [25].

### Survival analysis

Patient survival data were provided by the TCGA consortium. Survival analysis was performed using packages Surv and survminer (github.com/kassambara/survminer).

### RNA-binding protein motif detection

RNA-binding protein (RBP) motifs in position weight matrix format (PWM) were retrieved from the ATtRACT database (version 0.99β) [26], which contains 1196 motifs corresponding to 160 human RBPs. Sequences of 100 nt were extracted from the regions flanking retained and non-retained introns and scanned for the presence of motifs using the fimo tool provided by the meme suite [27].

## Results

### IR in breast tumours is reduced in contrast to high occurrence in normal breast tissue

**Fig. 1:**
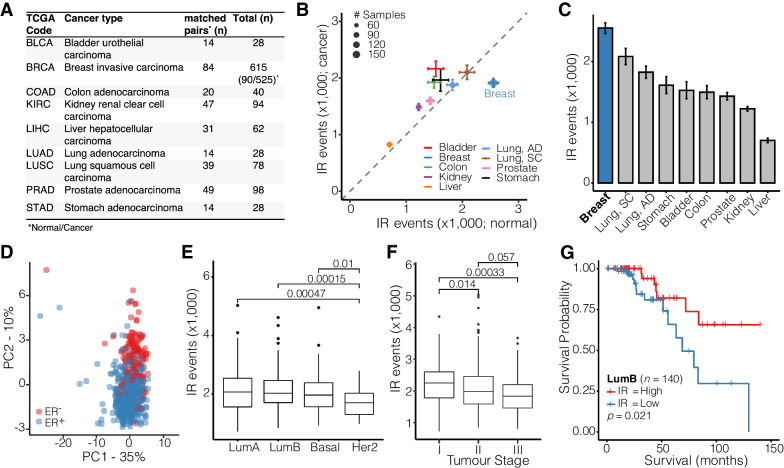
Breast cancer intron retention in the TCGA cohort. **A** Overview of TCGA samples analysed. **B** Scatter plot illustrating the average number of IR events in cancers vs adjacent normal tissues. Error bars indicate standard deviations. The size of the dots is proportional to the number of samples analysed. **C** Bar plot showing the average number of IR events in normal tissues in descending order. Error bars represent standard error of the mean. **D** PCA plot showing clusters of specific IR profiles in ER positive and ER negative tumour samples (*n* = 509). **E** Distributions of IR event frequencies in four major BrCa subtypes (LumA—Luminal A; LumB—Luminal B; Basal, HER2—human epidermal growth factor receptor 2 positive). **F** Distributions of IR event frequencies in tumour samples assigned to three tumour stages. **G** Kaplan–Meier plot illustrating the survival probabilities of Luminal B BrCa patients stratified by a high vs low number of IR events. Samples have been dichotomized based on the median number of IR events

To compare IR profiles across human cancers, we retrieved transcriptomics data for nine different solid tumours and matched adjacent normal tissues from The Cancer Genome Atlas (TCGA; Fig. 1A) and quantified IR using the IRFinder algorithm, which we have previously validated [17]. Overall, we identified a total of 11,943 unique IR events, of which 917 were shared among all nine cancers analysed.

Our analyses confirmed a previous report that BrCa is the only cancer in which the number of IR events is reduced compared to normal adjacent tissue (Fig. 1B) [28]. All other cancers exhibit increased IR compared to their matched adjacent normal tissue (Fig. 1B). However, we also noticed that the number of IR events in breast cancer itself was comparable with other cancers, while normal breast tissue presented with unusually high numbers of IR events (Fig. 1B). In fact, normal breast tissue had the highest IR frequencies compared to all other normal tissues (Fig. 1C). Therefore, reduced IR in tumours, which is unique to BrCa, can be largely attributed to normal breast tissue having the highest occurrence of IR events.

We applied beta regression models to identify differentially retained introns (dIRs) and found 3024 dIRs between normal and breast cancer (Additional file 1: Fig. S1A). Of these 210 were downregulated (in 160 genes) and 69 were upregulated (in 52 genes) with a ≥ 10% difference in the IR ratio (ΔIR ≥ 0.1). Downregulated IR events in BrCa are associated with processes related to cell cycle, nuclear division, and DNA replication among others (Additional file 1: Fig. S1B).

### High IR is associated with improved survival in Luminal B subtype breast cancer

Next, we explored whether the specific pattern of IR in BrCa is associated with clinical features. As shown in Additional file 1: Fig. S2A and Fig. 1D, IR patterns were distinct between BrCa vs normal tissues as well as oestrogen receptors positive (ER^+^) vs negative (ER^−^) samples, respectively. The human epidermal growth factor receptor 2 (HER2) amplified molecular subtype had the lowest average number of IR events (*n* = 1731) compared to the other three main subtypes Luminal A (*n* = 2089), Luminal B (*n* = 2113), and Basal (*n* = 2018) (Fig. 1E). Strikingly, HER2-amplified tumours are associated with a 2.9-fold increased hazard ratio (*p* = 0.001, Additional file 1: Fig. S2B). Moreover, advanced stage tumours (Stage III) had the lowest average number of IR events (*n* = 1913) compared to Stage II (*n* = 2064) and Stage I (*n* = 2210) tumours (Fig. 1F). Likewise, those tumours with the highest immunohistochemical (IHC) staining score for HER2 (score: 3) exhibited the lowest average number of IR events (*n* = 1825) compared to score 1 (*n* = 2095) or score 2 (*n* = 2137) tumours (Additional file 1: Fig. S2C). We also found that a high number of IR events is associated with better survival in patients with the Luminal B subtype (Fig. 1G; Additional file 1: Fig. S2D). Though, Luminal B is the only subtype where high IR is associated with a survival advantage (Additional file 1: Fig. S2E). Interestingly, the trend is reversed (although not significant) in Her2 positive breast tumours that do not belong to the luminal B subtype.

### Putative *trans*-regulators of IR in breast cancer

**Fig. 2:**
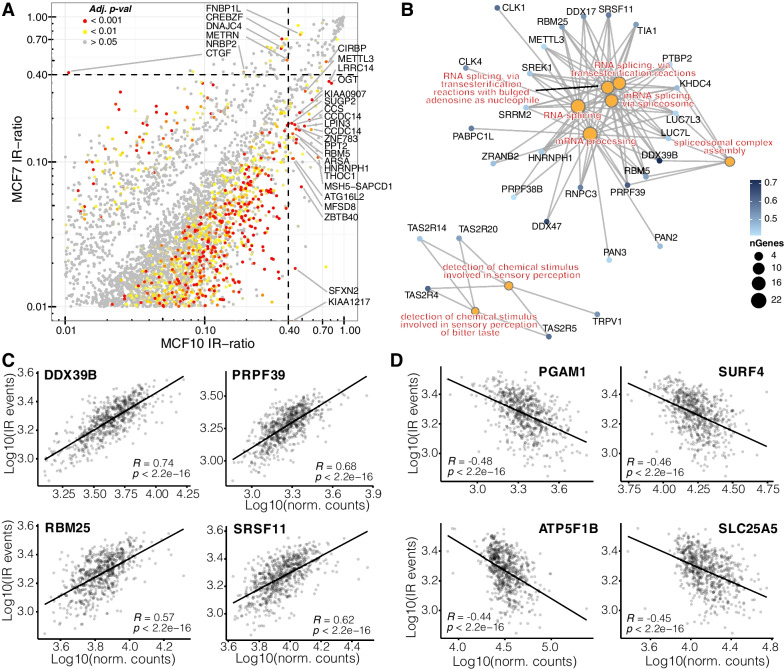
Putative regulators of intron retention in breast cancer. **A** Scatterplot showing differentially retained introns (dIR) between MCF7 and MCF10A cells. Genes associated with significant dIR events (*p*-adj. < 0.001) and IR ratios > 0.4 are labelled. Given the low number of replicates (*n* = 2), we have applied the Audic and Claverie test [29] for significance testing. **B** Enriched GO terms in genes positively correlated with IR. Circle size of the GO terms (orange) is denoted by the number of genes associated with them. **C** RNA splicing associated genes that most highly correlate with the number of IR events in each sample. The scatterplots illustrate the log10 number of IR events against the log10 normalized read counts for each gene. **D** Genes that most strongly anti-correlate with the number of IR events in each sample

To confirm that differences between tumour and normal breast tissue can be observed in vitro, we sequenced the transcriptomes of cultured ER^+^ MCF7 cells and non-tumorigenic MCF10A cells. Indeed, we observed a similar trend as in the TCGA cohort (Fig. 2A) and found that higher IR in the breast epithelial cell line (MCF10A) was associated with reduced gene expression (Additional file 1: Fig. S3). We also analysed sequencing data (Sequence Read Archive; SRA) of other cell lines representing each of the molecular subtypes (Additional file 1: Table S1). Comparing the number of IR events, we observed a similar trend as with the TCGA tumour samples, except for HER + HCC1419 cells, which have on average more IR events (though not significant) than cell lines of other subtypes (Additional file 1: Fig. S4).

To identify potential regulators of IR, we correlated IR frequencies in the TCGA-BRCA cohort with expression values (normalized RNA-seq counts) of ~ 23,000 genes (Additional file 2: Table S2). We performed Gene Ontology (GO) enrichment analysis on the top 5% genes with the highest (*r* > 0.41) and lowest (*r* < − 0.27) correlation coefficients to identify potential positive and negative regulators of IR, respectively. We found eight GO terms that were significantly enriched in positively correlated genes (*p*-adj. ≤ 0.05; Fig. 2B). Intriguingly, six of these GO terms were related to RNA splicing, with *DDX39B*, *RBM25*, *PRPF39,* and *SRSF11* being among the genes that most highly correlate with the number of IR events in each sample (Fig. 2C).

The four genes that most strongly anti-correlate with the number of IR events include the mutase PGAM1, the membrane protein encoding SURF4, the mitochondrial transmembrane transporter SLC25A5, and the mitochondrial ATP Synthase F1 Subunit Beta (ATP5F1B) (Fig. 2D). Strikingly, the top 10 most significant GO terms (out of 399) associated with genes that negatively correlate with the number of IR events correspond to mitochondrial processes and cellular energetics (Additional file 1: Fig. S5).

### Highly proliferating cells have fewer IR events

**Fig. 3:**
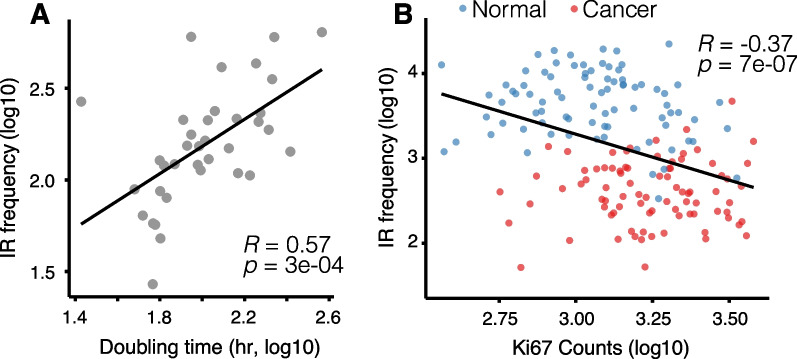
IR and cell proliferation. **A** Cancer Cell Line Encyclopedia (CCLE) cell doubling times (*x*-axis) correlate with number of IR events (*y*-axis). **B** Normalized read counts of proliferation marker Ki-67 anti-correlate with the number of IR events (*y*-axis). Red dots—tumour samples; blue dots—normal breast tissue

Next, we investigated whether changes in IR frequencies are associated with changes to cellular states. Since the energy demand of a cell is tightly coupled with proliferation, we sought to examine a potential link between doubling times of BrCa cells and the number of IR events in 36 BrCa-related cell lines of the Cancer Cell Line Encyclopedia (CCLE). Indeed, we found that cells with a slower doubling time exhibit a higher number of IR events (Fig. 3A). While this correlation is fairly robust, it should be noted that CCLE doubling times are an error-prone surrogate for cancer cell proliferation.

To corroborate this result, we also investigated whether a common proliferation marker would inversely correlate with IR frequencies. Since immunohistochemistry (IHC) staining of Ki-67 is unavailable for the TCGA cohort, we tested whether the proliferation rate in tissues might be estimated based on *MKI67* sequencing read counts. The *MKI67* gene encodes the proliferation marker protein Ki-67. Using Human Protein Atlas data, we confirmed that *MKI67* mRNA expression correlates with its Ki-67 protein staining intensities detected by IHC (Additional file 1: Fig. S6A). As expected, normalized *MKI67* read counts were higher in all nine cancers when compared to the respective adjacent normal tissues (Additional file 1: Fig. S6B). This suggests that *MKI67* read counts can be used as a proxy for IHC staining to estimate cellular proliferation rates. *MKI67* expression was also found to be inversely correlated to the doubling time of 36 CCLE cell lines (Additional file 1: Fig. S6C). We observed that the number of IR events in samples of the TCGA-BRCA cohort negatively correlated with the proliferation rate (Fig. 3B). However, no correlation was observed when analysing normal and tumour samples separately (Additional file 1: Fig. S7).

### The role of RNA-Binding Proteins in IR regulation

**Fig. 4:**
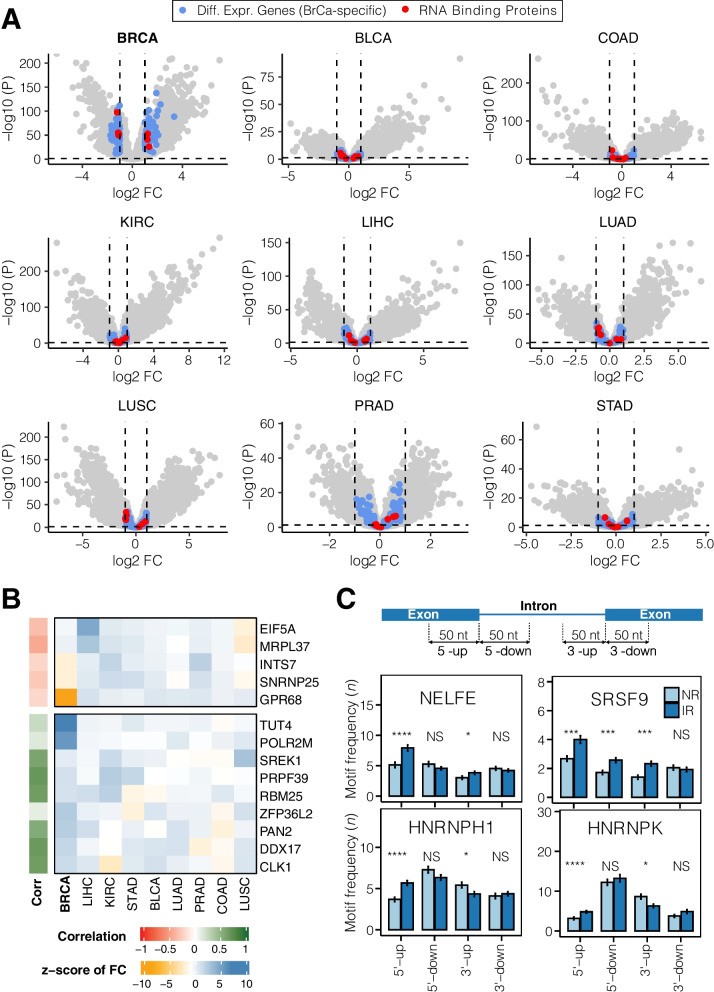
Breast cancer-specific gene expression and RBP analysis. **A** Volcano plots showing differentially expressed genes in nine tumour types vs adjacent healthy tissue. The dashed lines represent the *p* value cut-off (horizontal; *p* < 0.05) and fold change threshold (vertical |*FC*| ≥ 1). See Fig. 1A for cancer-type abbreviations. Highlighted in blue are genes that are exclusively differentially expressed in BrCa, while those in red represent RBPs within this subset. **B** Heatmap of genes specifically differently expressed in BrCa (represented by colour-coded *z*-score). Annotation bar (left) shows the colour-coded correlation coefficient between gene expression and number of IR events in each sample. **C** Bar plots show the frequencies with which known binding motifs occur around the splice sites (50 nt up-/downstream) of differentially retained (IR; dark blue) and non-differentially retained introns (NR; light blue). Differences in average frequencies were determined using Student’s *t* test. *p < 0.05, ***p < 0.001, ****p < 0.0001, NS—not significant

Next, we investigated genes that were specifically deregulated in BrCa. We identified a set of 150 genes that were *only* differentially expressed between BrCa and normal breast tissues, of which seven were RBPs (Fig. 4A). We calculated the *z*-score of each gene’s log fold change in BrCa versus the log fold change in other cancers in order to estimate the level of specificity of a gene being differentially expressed in BrCa only (Fig. 4B). Among the genes that are highly specifically over-expressed in BrCa are two known RBPs: *ZFP36L2* and *TUT4*. *ZFP36L2* promotes poly(A) tail removal of mRNA transcripts [30], while terminal uridylyl transferase 4 (*TUT4*) adds uridines to deadenylated transcripts [31]. Thus, both RBPs are mediators of mRNA decay, which could explain the observed reduction of IR transcripts in BrCa.

We also determined the frequency by which BrCa-specific genes occur in RNA-related gene sets (*n* = 138) in the Molecular Signatures Database (MSigDB; total ~ 5000 curated gene sets) [32]. While known RBPs such as *ZFP36L2* and *SNRNP25* (part of the minor U12-type spliceosome) are annotated in multiple RNA-related gene sets, other genes, that are specifically differentially expressed in BrCa, did not show any potential RNA-binding capabilities (Additional file 1: Fig. S8A).

In addition, we analysed differentially retained introns for occurrences of RBP binding motifs. We found that differentially retained introns were enriched in NELFE and SRSF9 binding sites in upstream exons (5'-up) and the 3' terminal region, respectively (Fig. 4C). Moreover, retained introns have fewer HNRNPH1 and HNRNPK binding sites in their 3' terminal region compared to non-retained introns (Fig. 4C; Additional file 1: Fig. S8B).

We conclude that RPBs are among the factors that facilitate reduced IR in BrCa by enabling efficient splicing of introns from pre-mRNA transcripts. However, RBPs specifically differentially expressed in BrCa are not among those with enriched binding motifs within and around differentially retained introns. This suggests that more complex, multifaceted regulatory mechanisms are causing the consistent reduction of IR in BrCa.

### Tissue composition affects cancer IR profiles

**Fig. 5:**
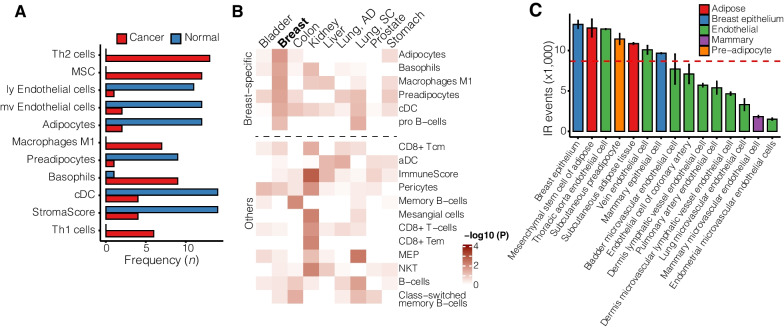
Breast tumour cell composition. **A** Frequently enriched cell types in breast tumours (red) and normal breast tissue (blue). **B** Heatmap illustrating cell-type enrichment in healthy adjacent tissue of nine TCGA cancer cohorts. **C** Abundance of IR events in purified cells. Colours indicate groups of cells belonging to the same family. Dashed red line represent mean number of IR events. Th1/2—T helper 1/2; MSC—mesenchymal stem cell; ly—lymphatic; mv—microvascular; a/cDC—activated/classical dendritic cell; Tcm—T central memory cell; Tem—T effector memory cell; NKT—natural killer T cell; MEP—megakaryocyte–erythroid progenitor cell. ImmuneScore quantifies the enrichment of an immune cell signature including B cells, T cells, DC, eosinophils, macrophages, monocytes, mast cells, neutrophils, and NK cells. StromaScore quantifies the enrichment of a stroma-type cell signature including adipocytes, endothelial cells, and fibroblasts

Since the reduction in IR events in BrCa contrasts with all other cancer types analysed, we examined a possible contribution from the changing cell composition in the tumour microenvironment compared to healthy breast tissue. Gene signature-based and machine learning-based algorithms have been developed to deconvolute the cell-type composition in bulk RNA-sequencing data [33]. To compare cell environmental profiles of TCGA breast tumour samples versus healthy adjacent control samples, we used the cell-type deconvolution algorithm xCell [34], which was trained on 1,822 pure human cell-type-specific transcriptomes extracted from single-cell transcriptome profiling data. xCell analysis revealed that the breast tumour cell composition is distinct from other cancers (Additional file 1: Fig. S9). Among the most enriched cell types in BrCa are T helper cells, mesenchymal stem cells, and basophils. These predictions are supported by recent single-cell BrCa profiling studies [35–37]. Normal breast tissue is enriched in endothelial cells, adipocytes, and dendritic cells (Fig. 5A).

Indeed, adipocyte and myeloid cell (M1 macrophages, basophils) enrichment are specific to normal breast tissue (Fig. 5B/C), which could explain the IR paradox in BrCa.

To determine whether cell types enriched in normal breast tissue have particularly high IR event frequencies, we retrieved RNA-sequencing data of 66 cell/tissue types from the ENCODE repository (Additional file 3: Table S3). Our analysis suggests that breast epithelial cells have the highest prevalence of IR followed by adipocytes (Fig. 5C), which could explain the drop in IR events in breast tumours.

## Discussion

IR is omnipresent in vertebrate species [2, 38] and affects up to 80% of human protein-coding genes [17]. Numerous studies have highlighted the functional importance of retained introns in a wide range of biological functions including cell differentiation and development [12, 39–42].

Since first reports in 2015 and subsequent confirmatory studies, BrCa has stood in stark contrast to other cancers concerning its burden of IR [28]. Dysregulation of *cis*- and *trans*-modulators can cause aberrant IR in various cancers [28]. For example, Dvinge et al*.* found that snRNA expression changes IR in the MCF7 cell line and to a certain degree in BrCa patient samples. They also showed that splicing factor knockdown can lead to increased IR in triple-negative BrCa (TNBC) [43]. Kim et al*.* found that some BrCa IR events anti-correlate with DNA methylation and that high IR levels in transcripts of migration and invasion inhibitory protein (MIIP) are associated with increased survival in European-American patients with invasive breast carcinoma [44].

We confirmed a consistent reduction of IR events in TCGA breast adenocarcinoma samples compared to adjacent normal breast tissue. While BrCa is the only cancer where this reduction is observed, IR frequencies are, in fact, comparable to those observed in other cancer types. This is due to the excessively large number of IR events in healthy breast tissue. Gascard et al*.* found that IR increases with differentiation state in normal human breast cells with fewer IR events in myoepithelial cells and seven times more events in luminal epithelial cells [45]. Indeed, our results suggest that an important factor in the reduction of IR events in breast tumours is the changing cell composition from adipocyte and epithelial cell-rich breast tissue to lymphocyte-infiltrated breast tumours. Adipocytes and epithelial cells have one of the highest IR frequencies in their transcriptomes compared to other cell types, while lymphocytes are known to have low IR counts [46]. Siang and co-workers have shown in this context that the RBP human antigen R (HuR), which is involved in pre-mRNA processing, is a negative regulator of adipogenesis [47]. Interestingly, Diaz-Muñoz et al*.* demonstrated that HuR binding to introns modulates alternative intron usage [48]. This may contribute to the high IR observed in adipocyte-rich normal breast tissue.

Aberrant IR has previously been associated with disease phenotypes and clinical outcomes. For example, IR in *CMYC* and *SESTRIN1* genes was shown to be a reliable molecular marker separating melanoma from non-melanoma tumours [14] and Sznajder and colleagues have shown that IR can be used as a biomarker in hereditary repeat expansion diseases [15]. Despite marked differences between tumour and normal breast tissue, IR profiles in our analysis also differ between ER^+^ versus ER^−^ tumours. The survival advantages associated with high IR numbers in the Luminal B subtype suggest that this form of alternative splicing should be considered for therapeutic exploitation. However, the exact mechanisms whereby dynamic IR profiles lead to differences in clinical outcomes would be the subject of future studies.

The inverse relationship between IR and cell proliferation has been previously observed in the context of B cell development and T cell activation [46, 49]. Our results demonstrate that the number of IR events positively correlates with longer cancer cell doubling times and that more IR events are associated with slower cell proliferation in BrCa. Our data show that HER2 positive breast tumours have the lowest number of IR events. HER2 is known to induce cell proliferation in human cancers and is associated with poor prognosis in BrCa [50]. These results suggest that IR is a mechanism that counteracts tumour growth and would provide opportunities as therapeutic targets. Interestingly, the tumour suppressor Herstatin, expressed in healthy breast tissue [51], is a splice variant of the oncogene *HER2*, with a retained intron 8 [52]. Herstatin is a secreted autoinhibitor of Her2 [52], and intron 8 retention is regulated by RBPs of the HNRNP1 family (including H1, D, and A2/B1) [53]. Koedoot and co-workers have demonstrated that inhibition of cell proliferation can be achieved via splicing factor knockdown in TNBC [54].

## Conclusions

In summary, our study sheds light on the unique causes and consequences of aberrant splicing in BrCa. The modulation of IR levels may offer novel opportunities for personalized BrCa treatment, especially in hormone- and chemotherapy-resistant subtypes.

## Supplementary Information

**Additional file 1.** Supplementary figures and table.

**Additional file 2.** Correlated analysis between IR frequencies and normalized gene counts from the TCGA-BRCA cohort.

**Additional file 3.** RNA sequencing data of 66 cell/tissue types from the ENCODE repository.

## Data Availability

RNA-sequencing data from MCF7 and MCF10 cells have been deposited at Gene Expression Omnibus (GSE196557). (https://www.ncbi.nlm.nih.gov/geo/query/acc.cgi?acc=GSE196557).

## Abbreviations

NR: Non-differentially retained introns
BrCa: Breast cancer
CCLE: Cancer Cell Line Encyclopedia
dIR: Differentially retained introns
ER: Oestrogen receptor
FDR: False discovery rate
HER2: Human epidermal growth factor 2
ID: Intron depth
IHC: Immunohistochemistry
IR: Intron retention
MSigDB: Molecular Signatures Database
NMD: Nonsense-mediated decay
PCA: Principal component analysis
PWM: Position weight matrix
RBP: RNA-binding protein
SE: Splice exact
SL: Splice left
SR: Splice right
TCGA: The Cancer Genome Atlas
TNBC: Triple-negative breast cancer
