# Supplementary material for: Towards resolution of the intron retention paradox in breast cancer
Source: Breast Cancer Res. 2022 Dec 29;24:100. doi: 10.1186/s13058-022-01593-1 (PMC9798573; doi:10.1186/s13058-022-01593-1)
Supplement: Supplementary file 1 — Additional file 1. Supplementary figures and table. [file 13058_2022_1593_MOESM1_ESM.docx]

# Supplementary Materials


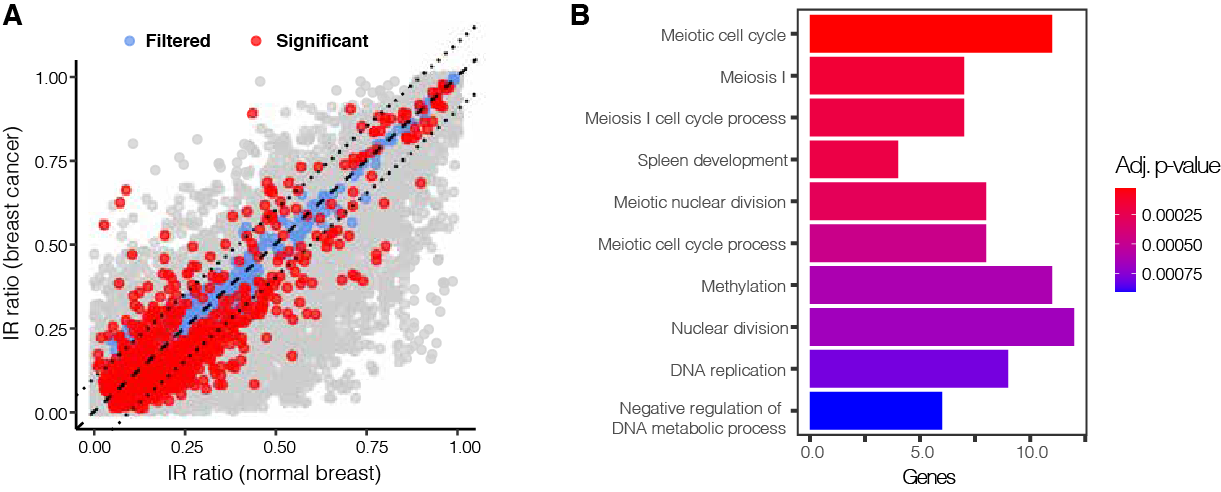


Supplementary Figure 1 Intron retention in breast cancer versus normal adjacent tissue. (A) IR ratios of differentially retained introns in BrCa and normal breast tissue (blue – filtered introns; red – significantly differentially retained introns). (B) Gene Ontology enrichment of genes with reduced IR in BrCa. The analysis is based on n = 150 matched samples.


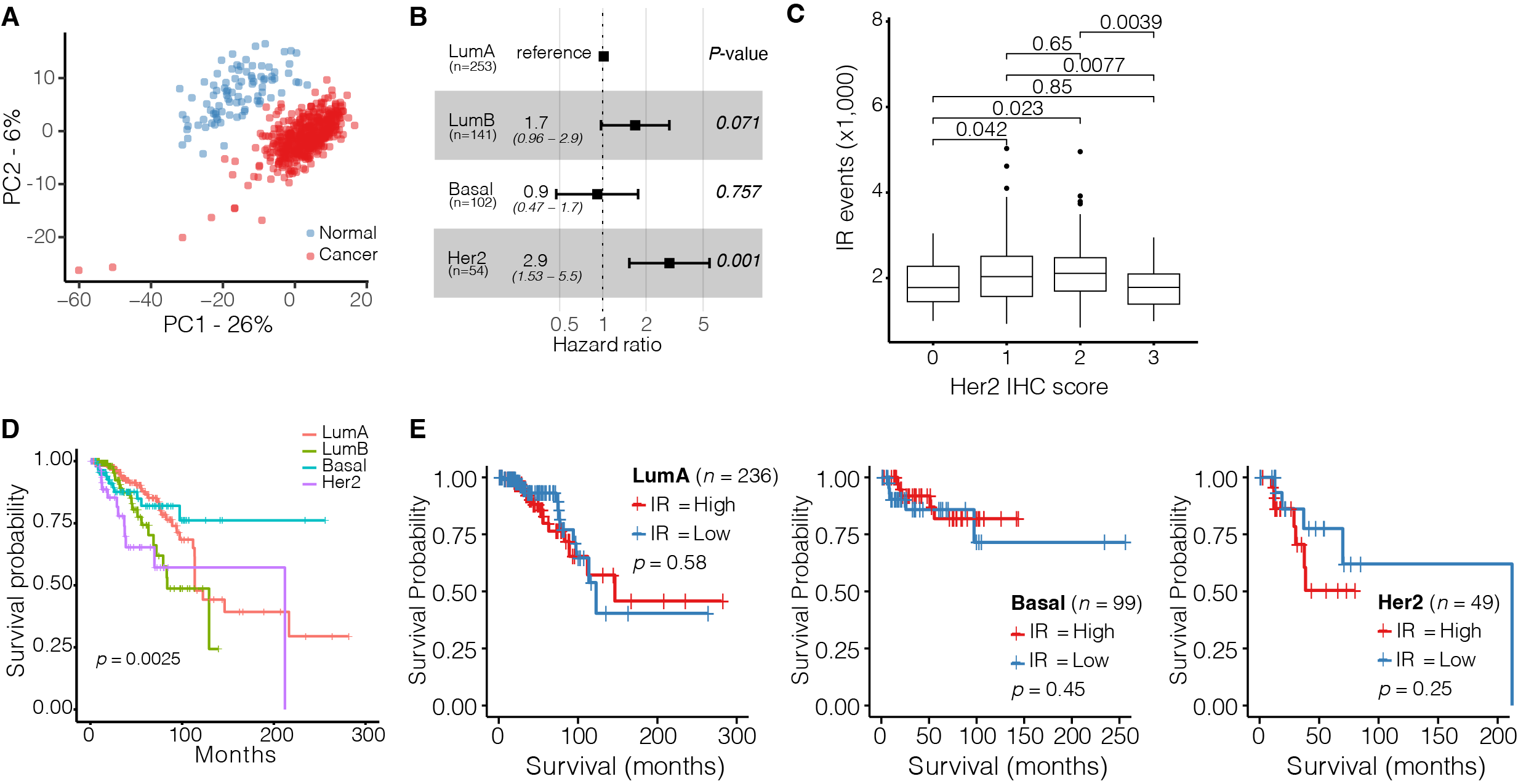


Supplementary Figure 2 Clinical relevance of IR in breast cancer. (A) Principal component analysis (PCA) plot illustrating distinct IR profiles of BrCa (red) and normal breast tissue (blue) samples (*n* = 615). (B) Cox hazard ratio for BrCa subtypes. Luminal A breast cancer was chosen as reference as it is the most benign subtype of BrCa and exhibits the greatest level of IR. (C) Distributions of IR event frequencies in tumour samples were assigned to each of the four HER2 immunohistochemistry (IHC) scores. (D) Kaplan-Meier plot showing the survival probability of BrCa patients with different molecular subtypes. (E) Kaplan-Meier plots comparing survival probabilities with high or low numbers of IR events in different BrCa subtypes.


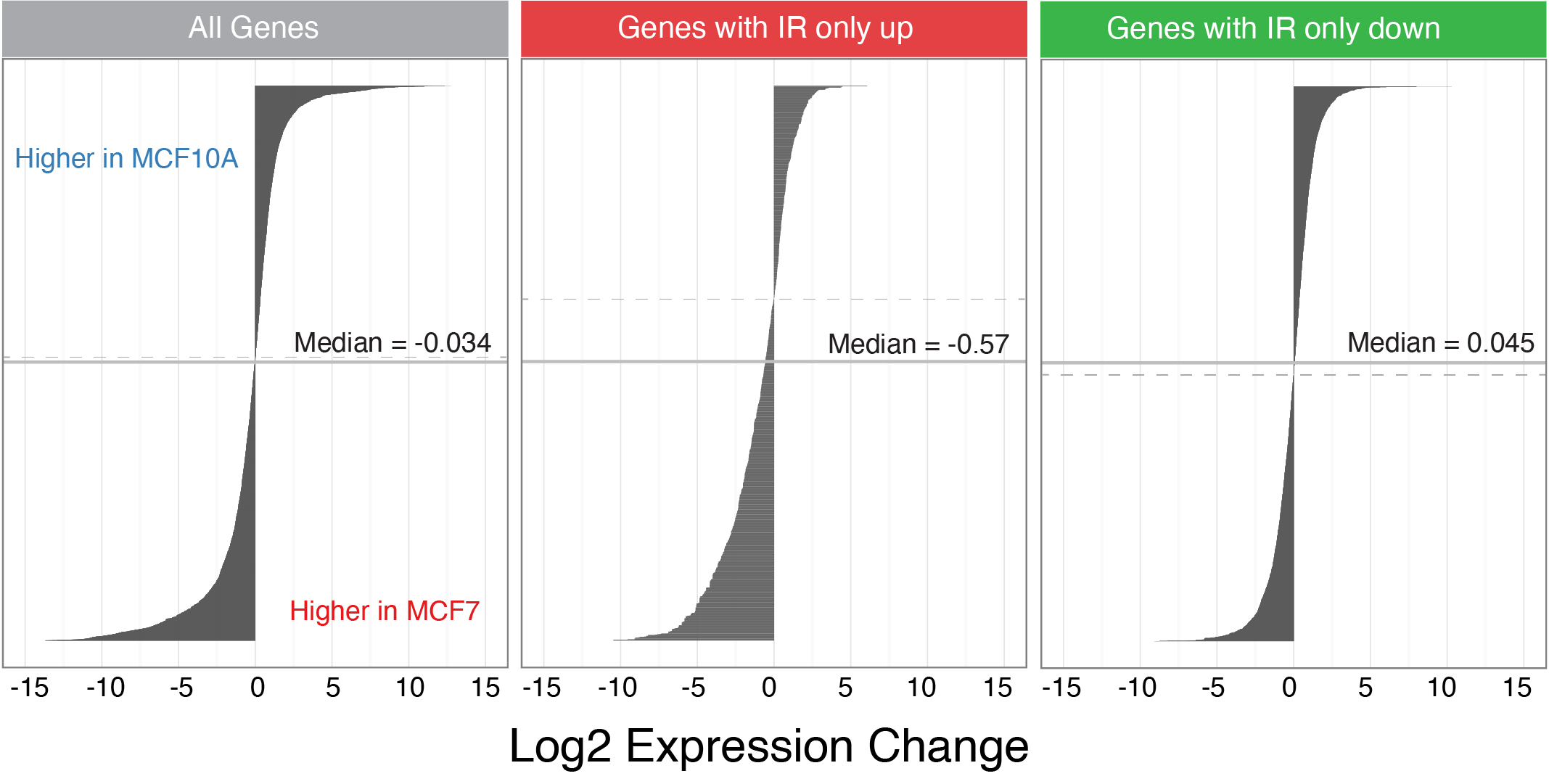


Supplementary Figure 3 Intron retention effects on gene expression. Waterfall plots showing the distribution of all up- and downregulated genes (left), those that have increased (middle) or decreased (right) IR levels in MCF10a vs MCF7. Dashed lines indicate inflection point.

| **Subtype** | **Cell Line** | **Replicates** | **Source** |
| --- | --- | --- | --- |
| Luminal A | MCF7 | 2 | in-house |
| Luminal B | BT474 | 3 | GSM5413113, GSM5413114, GSM5413115 |
| Basal | BT20 (TNA)  MDA-MB-231 (TNB) | 3/3 | SM5257949, SM5257950, SM5257951  GSM5413110, GSM5413111, GSM5413112 |
| Her2+ | HCC1419 | 3 | GSM4603165, GSM4603166, GSM4603167 |
| Normal | MCF10A | 2 | in-house |

Supplementary Table 1 Sources of mRNA sequencing data of cell lines representative of BrCa subtypes.


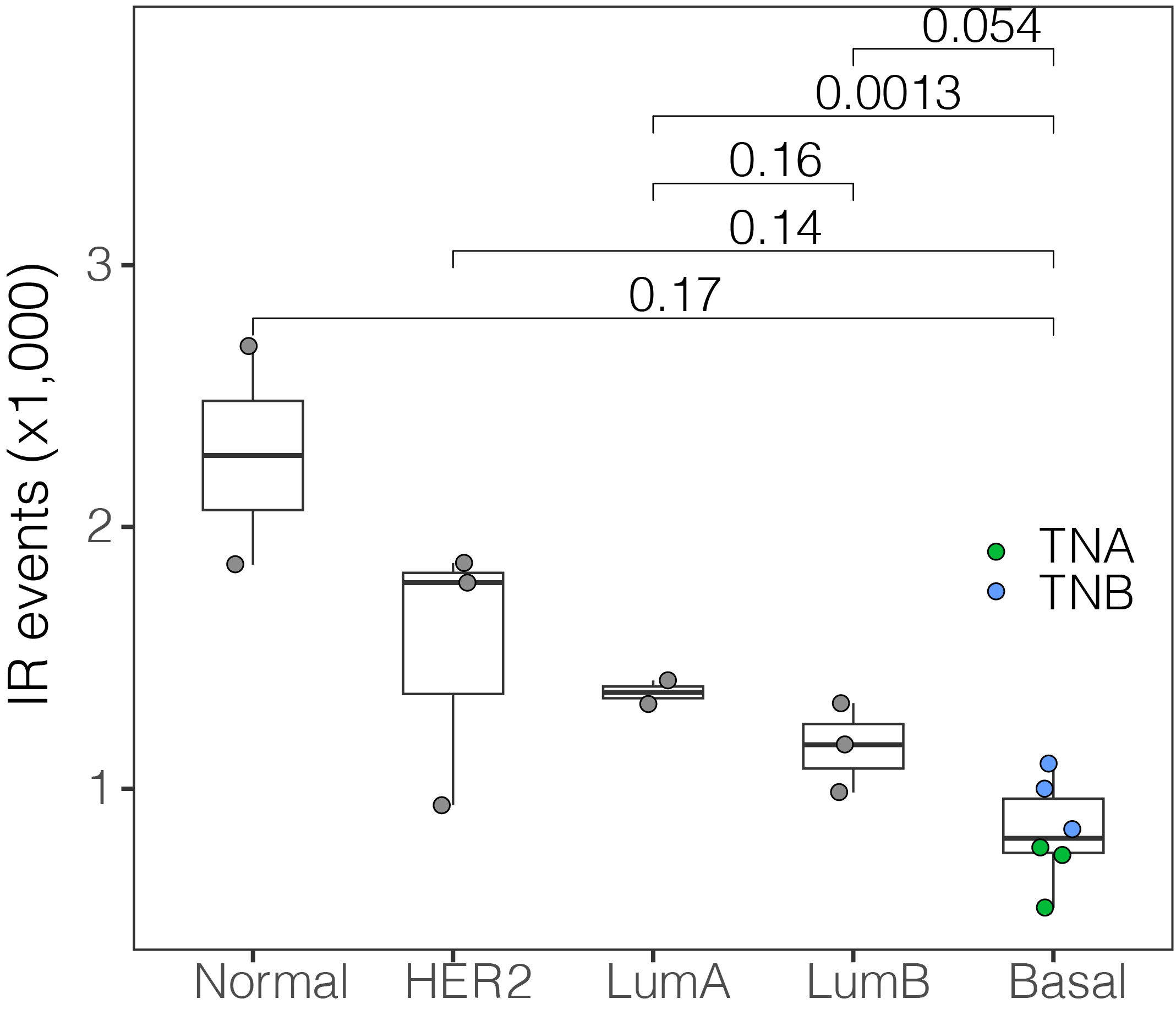


Supplementary Figure 4 IR event frequencies in cell lines representing four major BrCa subtypes. LumA – Luminal A (MCF7 cells); LumB –Luminal B (BT474 cells); TNA – triple negative BrCa A (BT20 cells); TNB triple negative BrCa B (MDA-MB-231 cells), HER2 - human epidermal growth factor receptor 2 positive (HCC1419 cells). Normal breast tissue represented by MCF10A cells. T-tests were used for pair-wise comparisons; p-values are indicated above each pair of subtypes.


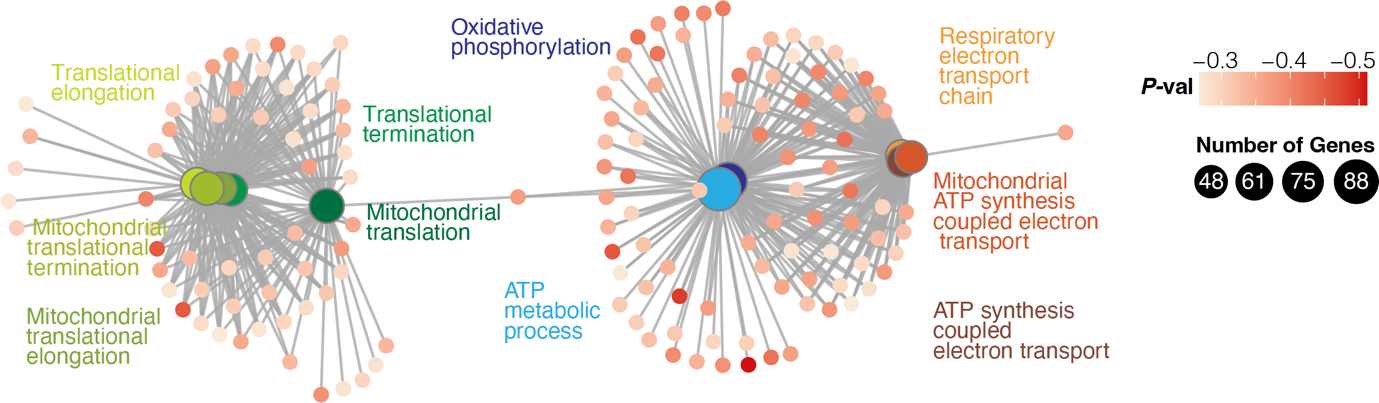


Supplementary Figure 5 Intron retention effects on gene expression. Top 10 most significant GO terms associated with genes that negatively correlated with the number of IR events.


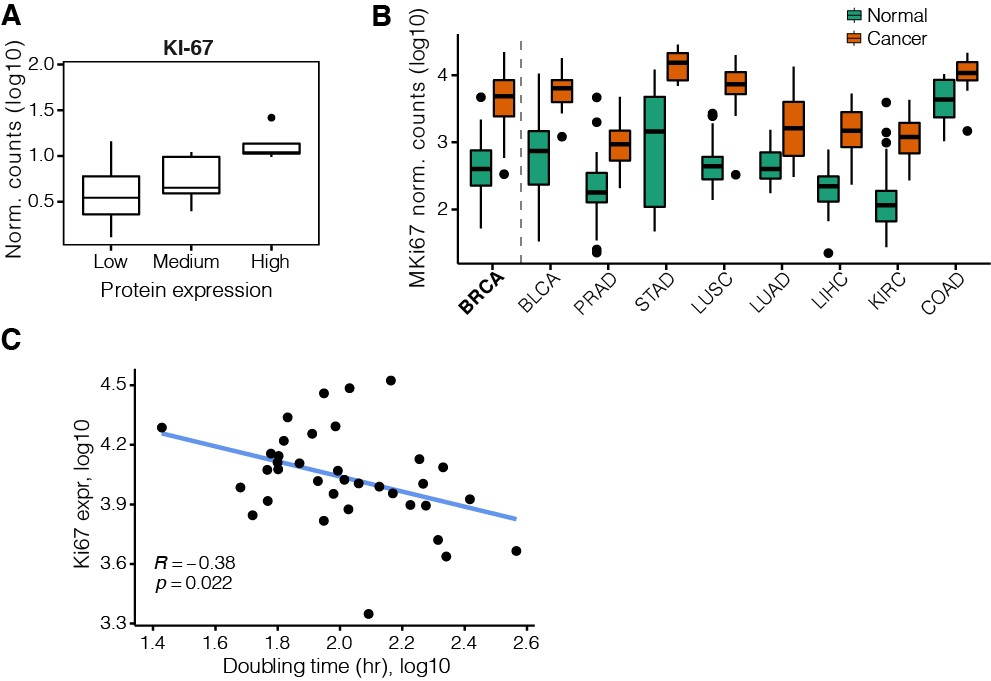


Supplementary Figure 6 Proliferation markers of cancer cells. (A) *MKI67* mRNA expression corresponds to Ki-67 protein levels based on immunohistochemistry data (Human Protein Atlas). Box-whisker plots represent log10 normalised counts of *MKI67* and *PCNA,* two cell proliferation marker genes. Horizontal lines indicate median count number. Dots represent outliers. (B) Normalized *Ki67* read counts used as proliferation index for nine TCGA tumour types. Dots represent outliers. (C) Anti-correlation of *MKI67* expression (normalised read counts; y-axis) and cell doubling time (x-axis) in CCLE BrCa cell lines.


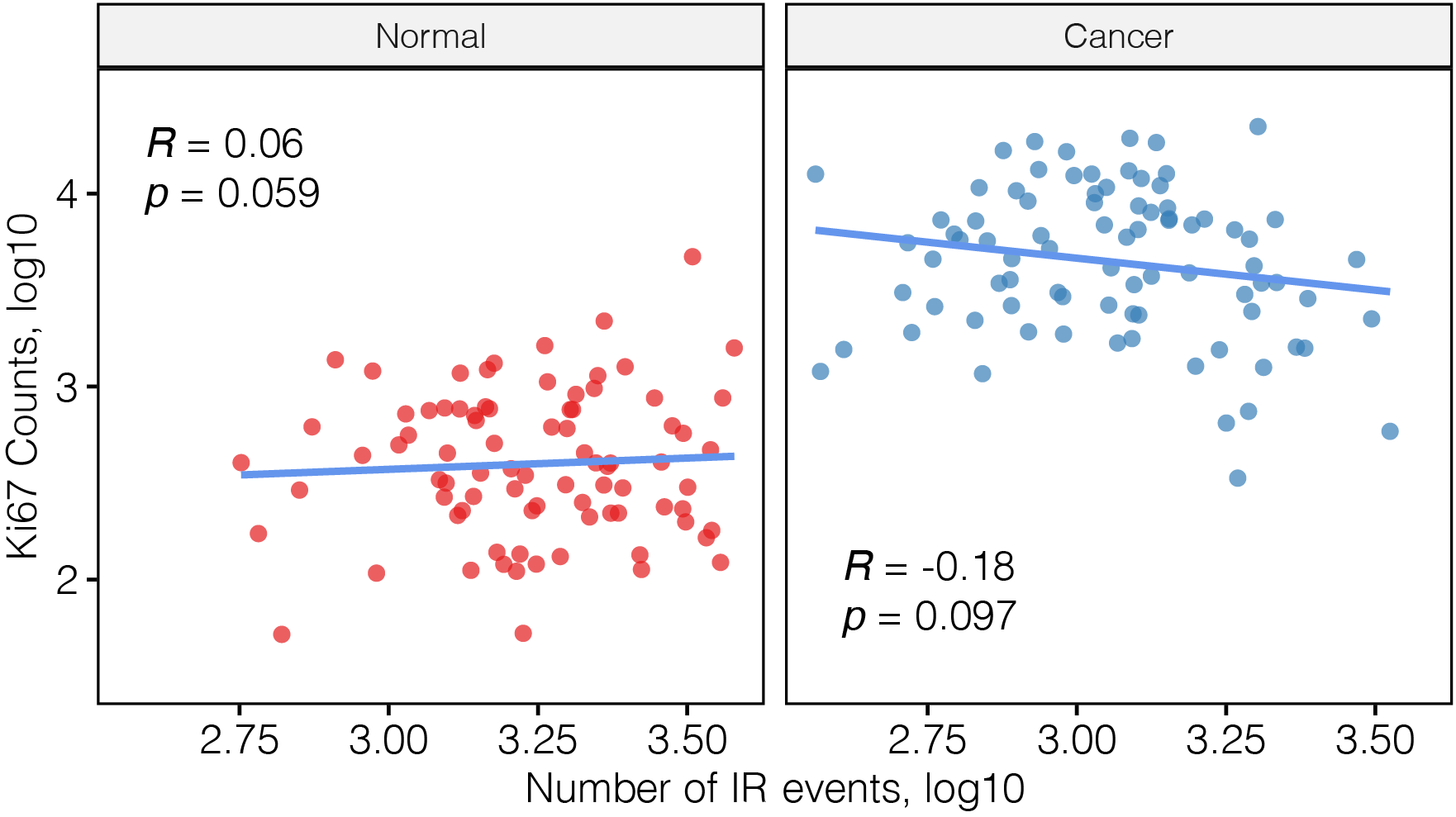


Supplementary Figure 7 IR and cell proliferation. Normalized read counts of proliferation marker Ki67 and number of IR events in normal (left) and tumour samples (right).


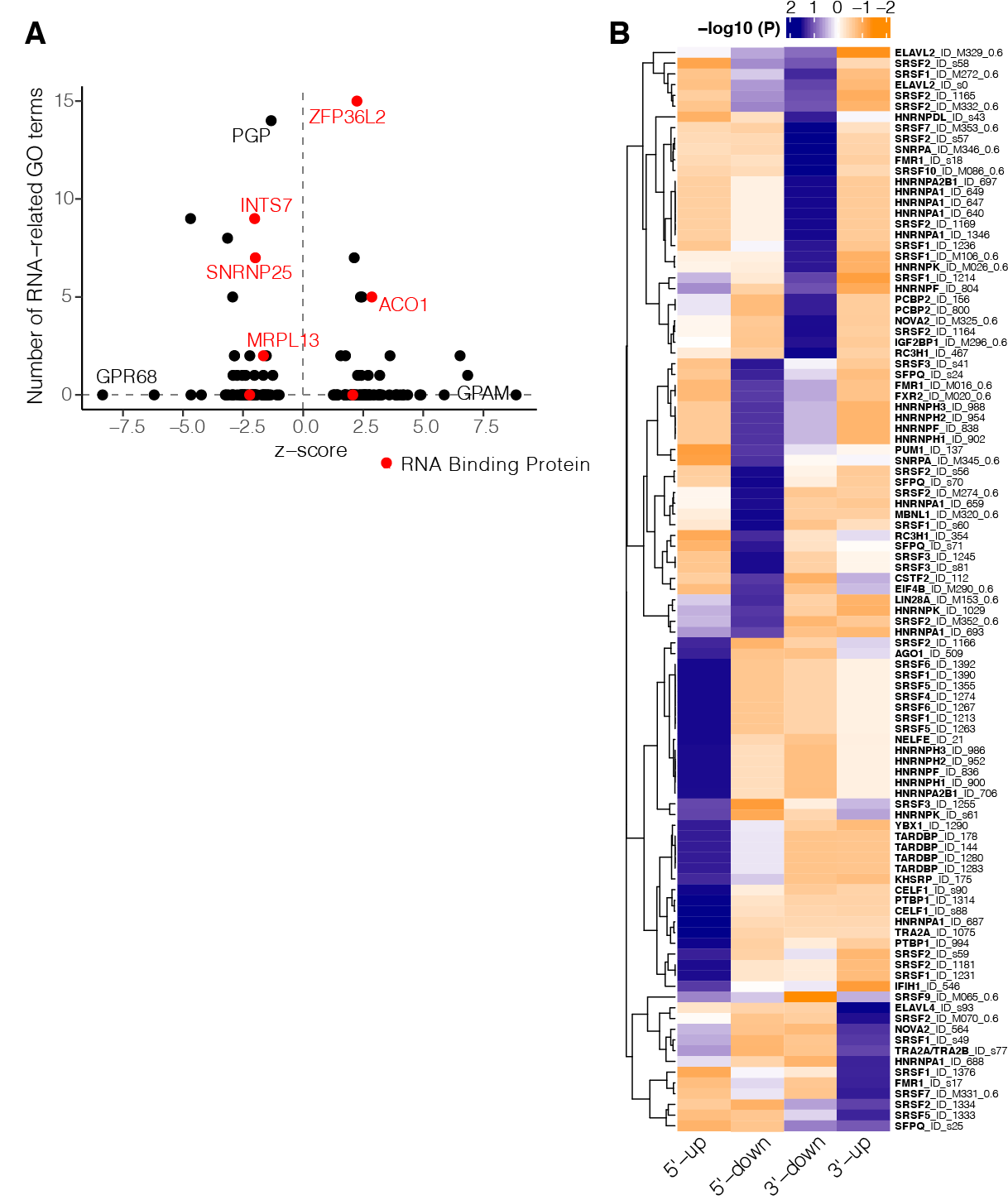


Supplementary Figure 8 RBP motif enrichment. (A) Frequency of BrCa-specific genes occuring in RNA-related gene sets (n = 138) in the Molecular Signatures Database. Highlighted in red are RNA binding proteins. (B) Heatmap of enriched RBP binding motifs.


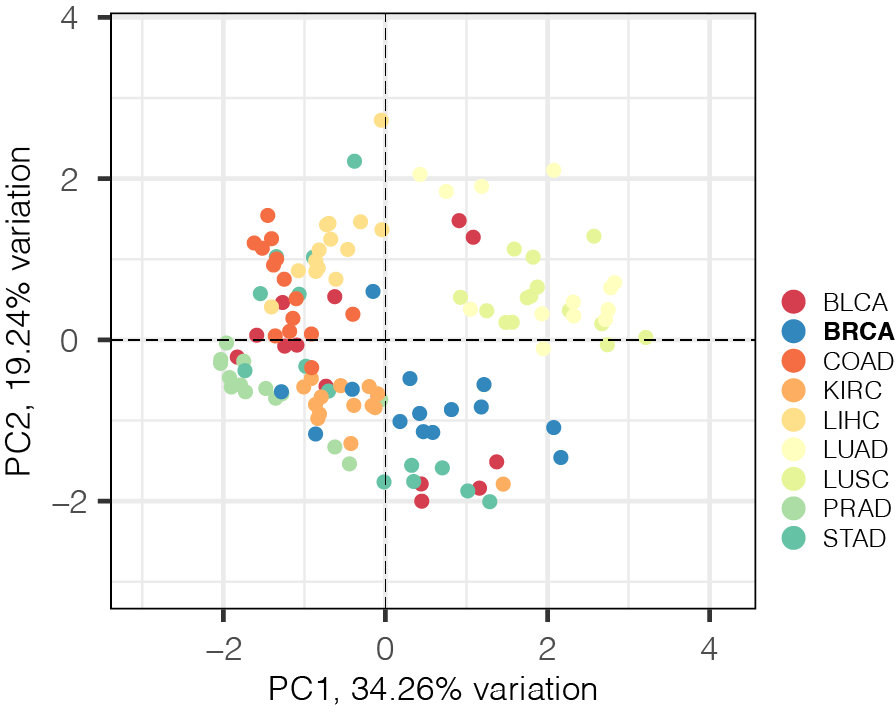


Supplementary Figure 9 Principal component analysis of tumour cell composition profiles.
